# Whole Genome DNA Methylation Analysis of Active Pulmonary Tuberculosis Disease Identifies Novel Epigenotypes: *PARP9*/*miR-505*/*RASGRP4*/*GNG12* Gene Methylation and Clinical Phenotypes

**DOI:** 10.3390/ijms21093180

**Published:** 2020-04-30

**Authors:** Yung-Che Chen, Chang-Chun Hsiao, Ting-Wen Chen, Chao-Chien Wu, Tung-Ying Chao, Sum-Yee Leung, Hock-Liew Eng, Chiu-Ping Lee, Ting-Ya Wang, Meng-Chih Lin

**Affiliations:** 1 Division of Pulmonary and Critical Care Medicine, Department of Medicine, Kaohsiung Chang Gung Memorial Hospital and Chang Gung University College of Medicine, Kaohsiung 83301, Taiwan; cchsiao@mail.cgu.edu.tw (C.-C.H.); my47104710@gmail.com (C.-C.W.); tychao@adm.cgmh.org.tw (T.-Y.C.); sumyeeleung@hotmail.com (S.-Y.L.); choupeen@gmail.com (C.-P.L.); filling.tw@yahoo.com.tw (T.-Y.W.); 2 Graduate Institute of Clinical Medical Sciences and Department of Medicine, College of Medicine, Chang Gung University, Taoyuan 33302, Taiwan; 3 Molecular Medicine Research Center, and Bioinformatics Center, Chang Gung University, Taoyuan 33302, Taiwan; afratw@gmail.com; 4 Institute of Bioinformatics and Systems Biology, National Chiao Tung University, Hsinchu 30068, Taiwan; 5 Department of Biological Science and Technology, National Chiao Tung University, Hsinchu 30068, Taiwan; 6 Center for Intelligent Drug Systems and Smart Bio-devices (IDS2B), National Chiao Tung University, Hsinchu 30068, Taiwan; 7 Division of Clinical Pathology, Kaohsiung Chang Gung Memorial Hospital and Chang Gung University College of Medicine, Kaohsiung 83301, Taiwan; eng4087@cgmh.org.tw

**Keywords:** pulmonary TB, whole genome DNA methylation, *PARP9*, *miR505*, *RASGRP4*, *GNG12*

## Abstract

We hypothesized that DNA methylation patterns may contribute to the development of active pulmonary tuberculosis (TB). Illumina’s DNA methylation 450 K assay was used to identify differentially methylated loci (DML) in a discovery cohort of 12 active pulmonary TB patients and 6 healthy subjects (HS). DNA methylation levels were validated in an independent cohort of 64 TB patients and 24 HS. Microarray analysis identified 1028 DMLs in TB patients versus HS, and 3747 DMLs in TB patients after versus before anti-TB treatment, while autophagy was the most enriched signaling pathway. In the validation cohort, *PARP9* and *miR505* genes were hypomethylated in the TB patients versus HS, while *RASGRP4* and *GNG12* genes were hypermethylated, with the former two further hypomethylated in those with delayed sputum conversion, systemic symptoms, or far advanced lesions. *MRPS18B* and *RPTOR* genes were hypomethylated in TB patients with pleural involvement. *RASGRP4* gene hypermethylation and RPTOR gene down-regulation were associated with high mycobacterial burden. TB patients with *WIPI2*/*GNG12* hypermethylation or *MRPS18B*/*FOXO3* hypomethylation had lower one-year survival. In vitro ESAT6 and CFP10 stimuli of THP-1 cells resulted in DNA de-methylation changes of the *PARP9*, *RASGRP4*, *WIPI2*, and *FOXO3* genes. In conclusions, aberrant DNA methylation over the PARP9/miR505/RASGRP4/GNG12 genes may contribute to the development of active pulmonary TB disease and its clinical phenotypes, while aberrant DNA methylation over the *WIPI2*/*GNG12*/*MARPS18B*/*FOXO3* genes may constitute a determinant of long-term outcomes.

## 1. Introduction

Tuberculosis (TB) is an airborne infectious disease that kills almost two million individuals every year worldwide [1]. Epigenetics of immune genes associated with TB susceptibility and immune defense mechanism remain largely untapped field for better TB control. Recent studies suggest that *Mycobacterium tuberculosis* (*Mtb*) can alter the host epigenome to modulate the transcriptional machinery by either activation or suppression of key immune genes involved in immune response or pathogen persistence [2,3].

DNA methylation, occurring at position 5 of the pyrimidine ring of cytosines in the context of the cytosine followed by guanine dinucleotide sequence (CpG) form in mammalians, is a heritable, tissue-specific, and reversible gene regulatory process that is highly modified in response to environmental factors, such as infection. Increased DNA methylation in promoter regions of human genes is associated with their transcriptional repression, while increased DNA methylation in gene bodies is generally associated with transcription activation [4,5]. Previous research shows that aberrant DNA methylation of the toll-like receptor 2, vitamin D receptor, and interleukin-18 genes as well as the tumor suppressor gene CD82, together with genetic polymorphisms, is associated to TB predisposition and can regulate host immune responses to *Mtb* [6,7,8,9,10]. A secreted *Mtb* protein, Rv2966c, has been demonstrated to be a 5-methylcytosine-specific DNA methyltransferase, interacting with host chromatin predominantly through non-CpG methylation and histone H3/H4 binding [11]. Two recent studies have shown that protective activity against mycobacterium correlates with altered DNA methylation pattern in immune cells from Bacillus Calmette-Guérin-vaccinated adults and infants, leading to the hypothesis that modulation of epigenetic programming of the cell to induce trained innate immunity may boost early clearance of *Mtb* upon infection [12,13,14].

The aim of this study is to improve the understanding of the susceptibility to active TB disease as well as the epigenetic mechanisms that regulate initial human immunologic responses or fine tune trained memory immunity related to *Mtb* persistence and dissemination by investigating the DNA methylation levels of genes in peripheral blood mononuclear cells (PBMCs) on a genome-wide scale. We hypothesized that the DNA methylation profiles of PBMCs in active pulmonary TB patients would be different from those in healthy subjects (HS), and that additional differences would be seen in those with certain clinical phenotypes and after completing anti-TB treatment, with the goal of identifying novel epigenetic changes related to early infection, disease progression, treatment response, and long-term outcomes of active pulmonary TB disease.

## 2. Results

### 2.1. Demographics of the Participants

The characteristics and biochemistry data of both the discovery and validation cohorts are listed in Table 1. The study population was all residents in Taiwan. Age, male gender ratio, smoking history, alcoholism history, and co-morbidities were all matched between the case and control groups.

### 2.2. Whole Genome DNA Methylation Profiles of the Discovery Cohort

**Figure 1:**
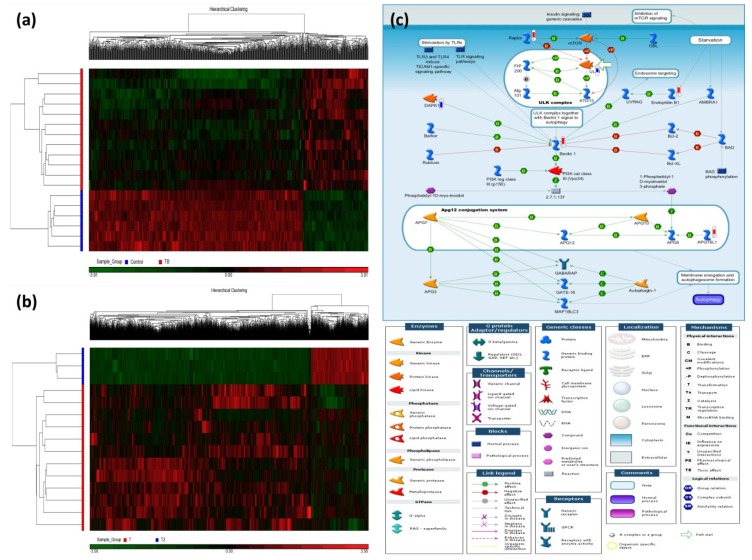
Heatmaps of the differentially methylated loci (DML) for the two comparisons and the most enriched pathway in the discovery cohort. Hierarchical clustering of DML in 21 samples classified into two comparisons: (**a**) Active TB patients versus healthy subjects (HS) and (**b**) TB patients after 6-month anti-TB treatment versus before treatment. (**c**) Autophagy-related gene signaling pathway enriched in active TB patients of the discovery cohort. In comparison between TB patients and healthy subjects, the significantly hypermethylated genes were highlighted with a red-colored barometric bar, while hypomethylated genes in a blue-colored bar. The changes represent the differences between the median M-values of HS and active TB patients. For example, the median M-value for HS and TB patients is 0.027 and 0.041, respectively, for *BECN1* (Beclin 1), indicating a higher methylation level in active TB.

Figure 1a shows two dimensional hierarchical clustering of the TB patients versus the HS, in which 1028 differentially methylated loci (DMLs) were identified (comparison I: 241 hypermethylation DMLs and 787 hypomethylated DMLs, all *p* values < 0.0005, all *q* values < 0.5). Table 2 lists top DMLs in comparison I. Figure 1b shows two dimensional hierarchical clustering of the TB patients after versus before 6-month anti-TB treatment, in which 3747 DMLs were identified (comparison II: 1298 hypermethylated DMLs and 2449 hypomethylated DMLs with treatment, all *p* < 0.005). Table S1 lists top DMLs in comparison II. The intersection of comparisons I and II resulted in 36 DMLs, among which 17 DMLs show reversion after treatment (9 hypermethylated DMLs: *C19orf69*, *MRPS18B*, *PGBD5*, *CCDC3*, *TBC1D16*, *LTV1*, *C5orf20*, *INPP5A*, *DNAH17*; 8 hypomethylated DMLs: *PPP2R2D*, *UBR1*, *TMPRSS9*, *HYAL2*, *MYH15*, *CNTLN*, *FAIM3*, *SLC38A10*).

### 2.3. Enrichment Pathway Analysis of the Differentially Methylated Genes

The top-ranking pathways enriched in comparison I included autophagy (Figure 1c), bile acids regulation of glucose/lipid metabolism via farnesoid X receptor (FXR), and cell adhesion of ephrin signaling (Table S2). The top-ranking pathways enriched in comparison II included activation of PKC via G-protein coupled receptors, NF-AT signaling, and ACM regulation of nerve impulse (Table S3).

### 2.4. Differential DNA Methylation Levels of the PARP9, MIR505, RASGRP4, and GNG12 Genes in Active TB Patients Versus HS in the Validation Cohort

**Figure 2:**
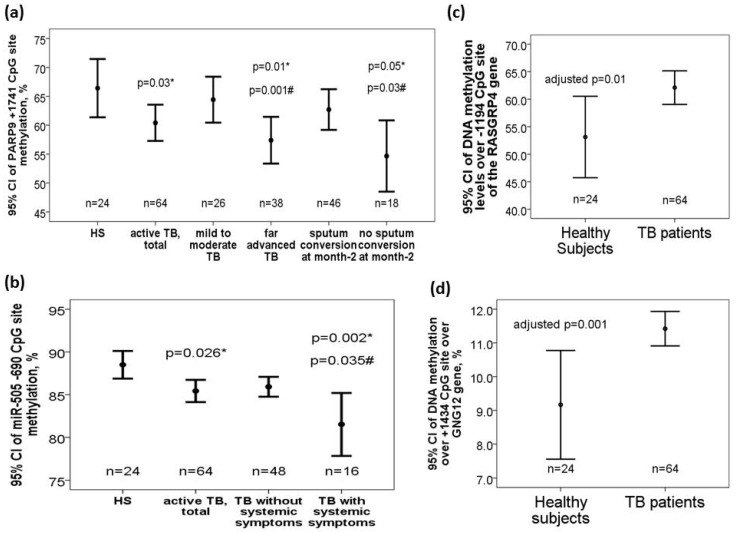
Differential DNA methylation levels over the *PARP9*, *miR-505*, *RASGRP4*, and *GNG12* genes in the validation cohort. (**a**) *PARP9* (+1741 CpG site) gene was hypomethylated in TB patients and further hypomethylated in those with far advanced lesions on CXR or without sputum smear/culture conversion to negative after 2-month anti-TB treatment. (**b**) *miR-505* (−690 CpG site) gene was hypomethylated in TB patients, and further hypomethylated in those with systemic symptoms. (**c**) *RASGRP4* (−1194 CpG site) and (**d**) *GNG12* (+1434 CpG site) genes were both hypermethylated in TB patients versus HS. *compared with HS and adjusted by multivariate linear regression analysis. #compared with TB patients without specific clinical phenotype and adjusted by multivariate linear regression analysis.

DNA methylation levels over +1741 CpG site of the *PARP9* gene (60.04 ± 12.7 versus 66.42 ± 11.95 %, adjusted *p* = 0.03) were decreased in TB patients versus that in HS. Subgroup analysis showed that DNA methylation levels over +1741 CpG site of the *PARP9* gene was further decreased in TB patients with far advanced lesions on CXR versus that in those with mild to moderate lesions on CXR (56.91 ± 12.92 versus 64.62 ± 11.07 %, adjusted *p* = 0.001) or HS (adjusted *p* = 0.01). DNA methylation levels over +1741 CpG site of the *PARP9* gene was also further decreased in TB patients without sputum AFB smear/*Mtb* culture conversion to negative after 2-month anti-TB treatment versus that in those with sputum conversion to negative at month 2 (54.72 ± 14.91 versus 62.29 ± 11.17 %, adjusted *p* = 0.03) or HS (adjusted *p* = 0.05) (Figure 2a). Both −696/−690 CpG sites of the *miR-505* gene (−696 CpG site: 73.17 ± 9.59 versus 77.21 ± 8.21 %, adjusted *p* = 0.06; −690 CpG site: 85.76 ± 5.21 versus 88.5 ± 3.83, adjusted *p* = 0.012) were decreased in TB patients versus that in HS. Subgroup analyses showed that DNA methylation levels over −690 CpG site of the *miR-505* was further decreased in TB patients with systemic symptoms (defined as fever or body weight loss) versus that in either those without systemic symptoms (83.5 ± 6.77 versus 86.51 ± 4.41, adjusted *p* = 0.035) or HS (adjusted *p* = 0.002) (Figure 2b). DNA methylation levels over -1194 CpG site of the *RASGRP4* gene (62.1 ± 13.8 versus 53.1 ± 11.5%, adjusted *p* = 0.01, Figure 2c) as well as over +1434 CpG site of the *GNG12* gene (11.4 ± 1.9 versus 9.1 ± 3.8%, adjusted *p* = 0.001, Figure 2d) were increased in TB patients at diagnosis versus HS.

### 2.5. Association of Aberrant DNA Methylation Levels of the GNG12, WIPI2, MRPS18B, and FOXO3 Genes with One-Year Survival in TB Patients in the Validation Cohort

**Figure 3:**
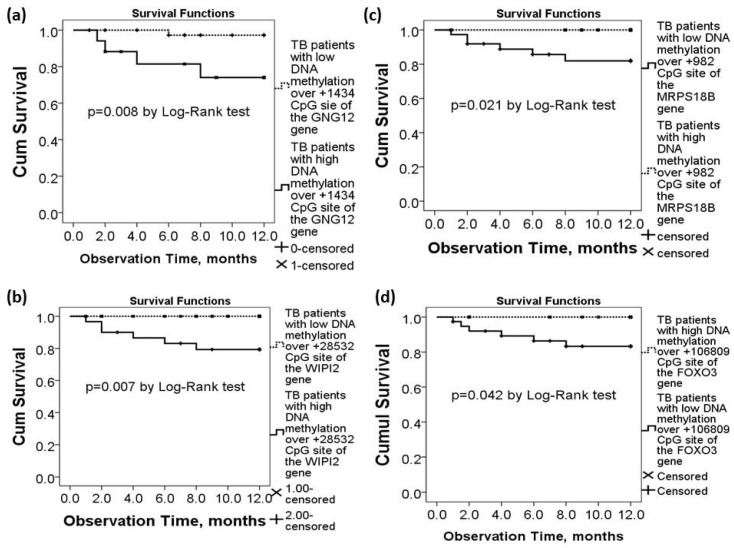
Differential DNA methylation levels of the *WIPI2*, *MRPS18B*, *FOXO3*, and *GNG12* genes in association with long-term outcomes of the active TB patients in the validation cohort. (**a**) TB patients with high DNA methylation levels over the *GNG12* (+1434 CpG site) gene had lower 1-year survival. Lower one-year survival rate was also noted in TB patients with (**b**) high DNA methylation levels over the *WIPI2* (+28532 CpG site) gene, (**c**) low DNA methylation levels over the *MRPS18B* (+982 CpG site) gene, or (**d**) low DNA methylation levels over the *FOXO3* (+106809 CpG site) gene.

TB patients with high DNA methylation levels over +1434 CpG site of the *GNG12* gene (>5%, *n* = 17) had lower one-year survival (all-cause mortality rate 23.5% versus 2.5%, *p* = 0.008 by Log-Rank test, Figure 3a) than those with low methylation levels (≤5%, *n* = 47). TB patients with high DNA methylation levels over +28532 CpG site of the *WIPI2* gene (>20%, *n* = 28) had lower one-year survival (all-cause mortality rate 17.85% versus 0%, *p* = 0.007 by log-rank test, Figure 3b) than those with low methylation levels (≤20%, *n* = 36). TB patients with low DNA methylation levels over +982 CpG site of the *MRPS18B* gene (<48%, *n* = 35) had lower one-year survival (all-cause mortality rate 14.28% versus 0%, *p* = 0.021 by log-rank test, Figure 3c) than those with high methylation levels (≥48%, *n* = 29). TB patients with low DNA methylation levels over +106809 CpG site of the *FOXO3* gene (<97%, *n* = 39) had lower one-year survival (all-cause mortality rate 15.38% versus 0%, *p* = 0.042 by log-rank test, Figure 3d) than those with high methylation levels (≥97%, *n* = 25).

### 2.6. Aberrant DNA Methylaion Levels of the MRPS18B, RASGRP4, and RPTOR Genes in TB Patients with High Bacterial Burden or Pleural Involvement

**Figure 4:**
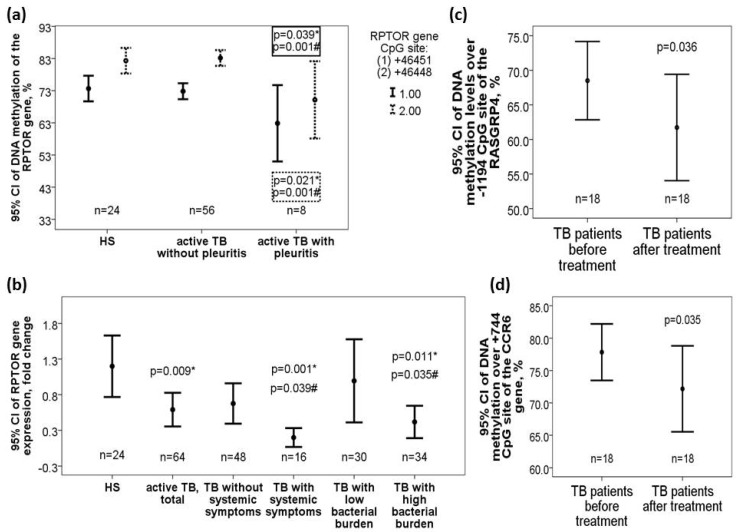
Differential DNA methylation levels of the *RPTOR*, *CCR6*, and *RASGRP4* genes along with corresponding *RATOR* gene expression changes in the validation cohort. (**a**) *RPTOR* gene (+46451/+46468) was hypomethylated in active pulmonary TB patients with pleurisy versus those without pleurisy or HS. (**b**) *RPTOR* gene expression was decreased in TB patients and further decreased in those with high bacterial burden or systemic symptoms versus those without the phenotype or HS. DNA methylation levels over the (**c**) *RASGRP4* (−1194 CpG site) genes and (**d**) *CCR6* (+744 CpG site) were both decreased after 6-month anti-TB treatment. *Compared with HS and adjusted by multivariate linear regression analysis. #Compared with TB patients without specific clinical phenotype and adjusted by multivariate linear regression analysis

DNA methylation levels over +982 CpG site of the *MRPS18B* gene (37.14 ± 9.14 versus 45.27 ± 9.86, adjusted *p* = 0.003 for TB patients with versus without pleuritis) and +46451/+46448 CpG sites of the *RPTOR* gene (+46451 CpG site: 62.86 ± 12.83 versus 72.85 ± 9.26%, adjusted *p* = 0.01; +46448 CpG site: 70.14 ± 12.98 versus 83.17 ± 9.25%, adjusted *p* = 0.001) were decreased in TB patients with pleuritis versus that in those without pleuritis or HS (+982 CpG site of the *MRPS18B* gene: adjusted *p* = 0.043; +46451 CpG site of the *RPTOR* gene: adjusted *p* = 0.039; +46448 CpG site of the *RPTOR* gene: adjusted *p* = 0.021) (Figure 4a). *RPTOR* gene expression was also decreased in TB patients versus HS (0.54 versus 1.15 fold change, adjusted *p* = 0.009), and further decreased in those with high bacterial burden (defined as sputum AFB ≥ 2+; 0.36 versus 0.94 fold change, adjusted *p* = 0.035) or systemic symptoms (0.15 versus 0.63 fold change, adjusted *p* = 0.039) (Figure 4b). DNA methylation levels over −1188 CpG site of the *RASGRP4* gene was increased in TB patients with high bacterial burden versus that in those with low bacterial burden (50.4 ± 10.7 versus 42.85 ± 10.8%, adjusted *p* = 0.03) or HS (43.8 ± 17.1%, adjusted *p* = 0.031).

### 2.7. Reversion of Altered DNA Methylation Levels of the CCR6 and RASGRP4 Genes in Active TB Patients after Anti-TB Treatment in the Validation Cohort

DNA methylation levels over both −1194 (61.7 ± 15.4 versus 68.5 ± 11.3%, *p* = 0.036, Figure 4c) and −1188 (49.9 ± 13.3 versus 57.1 ± 9.9%, *p* = 0.03) CpG sites of the *RASGRP4* gene as well as over +744 CpG site (72.1 ± 13.3 versus 77.8 ± 8.8%, *p* = 0.035, Figure 4d) of the CCR6 gene were reduced after 6-month anti-TB treatment versus that at diagnosis.

### 2.8. Effects of In Vitro Mtb-Specific Antigen Stimuli on DNA Methylation Levels and Gene Expressions of the 9 Candidate Genes

**Figure 5:**
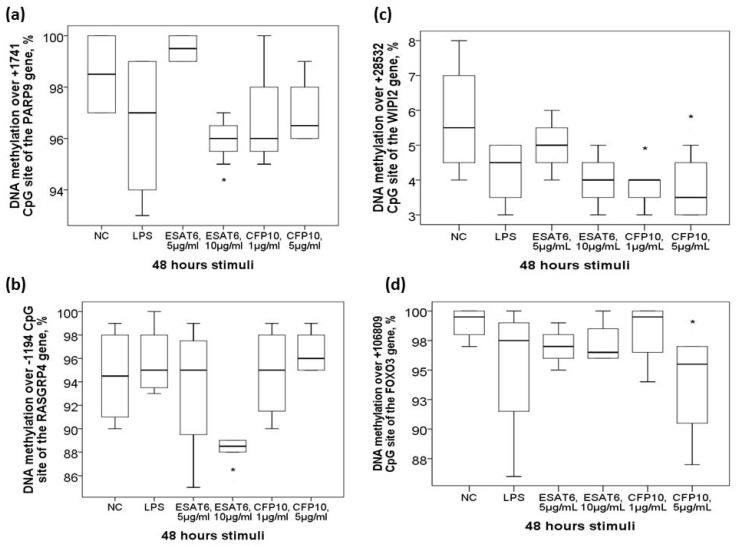
Aberrant DNA methylation levels of the candidate genes in response to in vitro mycobacterium tuberculosis-specific antigen stimuli. In vitro treatment of the THP-1 cells with ESAT-6 resulted in decreased DNA methylation levels over (**a**)+1741 CpG site of the *PARP9* gene and (**b**) -1194 CpG site of the *RASGRP4* gene. In vitro treatment of the THP-1 cells with CFP-10 resulted in decreased DNA methylation levels over (**c**) +28582 CpG site of the *WIPI2* gene and (**d**) +106809 CpG site of the *FOXO3* gene. *Compared between normal control (NC) and Mtb-specific antigen stimuli by Mann-Whitney U-test.

To determine whether *Mtb*-specific antigen per se can affect DNA methylation levels or corresponding gene expressions of the candidate genes, human monocytic THP-1 cells were exposed in vitro to either ESAT6, CFP10, LPS, or medium for 48 h. DNA methylation levels over +1741 CpG site of the PARP9 gene and −1194 CpG site of the *RASGRP4* gene were both decreased in response to ESAT6 treatment (both *p* values <0.05, Figure 5a,b). DNA methylation levels over +28532 CpG site of the *WIPI2* gene and +106809 CpG site of the *FOXO3* gene were both decreased in response to CFP10 treatment (both *p* values <0.05, Figure 5c,d). DNA methylation levels of the other 5 candidate genes all showed no significant changes. Gene expressions of the 9 candidate genes were all up regulated with either ESAT6 or CFP10 stimuli except for the *miR-505* gene, which showed down-regulation (Figure S1).

## 3. Discussion

In this study, we identified and replicated a specific association between active TB disease and *PARP9*/*miR-505* hypomethylation or *RASGRP4*/*GNG12* hypermethylation, as well as between clinical phenotypes of active TB and *MRPS18B*/*RPTOR* hypomethylation in PBMC DNA from patients with active pulmonary TB and HS. Moreover, we found a specific association of aberrant DNA methylation levels over the *WIPI2*, *MRPS18B*, *FOXO3*, and *GNG12* genes with lower one-year survival in TB patients, and a specific DNA de-methylation change of the *PARP9*, *RASGRP4*, *WIPI2*, and *FOXO3* genes in response to in vitro *Mtb*-specific antigen stimuli. Although some preliminary reports are available on blood DNA methylation changes of several genes in relation to the development of active TB disease, this is the first study to perform a large-scale analysis with replication of the principal finding [6,7,8,15,16]. In line with the recent findings that active de-methylation of thousands of CpG sites overlapping distal enhancer elements occurred during in vitro infection of human dendritic cells with *Mtb* [17], the majority of active TB-related DML identified in our study were hypomethylated. Likewise, global DNA hypomethylation of blood was reported in pediatric patients with active TB disease [18].

There are four adaptive or innate immunity-related genes identified in this study. Poly(ADP-ribose) polymerase family member 9 (PARP9) ubiquitin ligase can target host histone H2BJ and viral 3C protease to promote interferon-stimulated gene expression and regulate macrophage activation [19,20,21]. We speculate that hypomethylation over the *PARP9* gene body may contribute to lower innate immunity against *Mtb* and result in greater disease severity. RAS guanyl releasing protein 4 (RASGRP4) is required for the activation of both T cell and neutrophil, and for mast cells and dendritic cells to optimally induce acute natural killer cell-dependent interferon-gamma production in response to LPS [22,23,24]. We speculate that the *RASGRP4* gene is up-regulated in early immunological responses independent of its DNA methylation changes, and its hypermethylation over the promoter region at the later stage may contribute to counteracting the early immunological responses. *FOXO3* gene has been replicated consistently across diverse human populations for association with attainment of extreme old age [25]. Forkhead box O3 (FOXO3) inhibits cytokine production, opposes NF-κB activation, suppresses T-cell activation/proliferation, induces the synthesis of antimicrobial peptides, and lowers inflammation [26]. Furthermore, FOXO3 can reverse the inhibitory effect of miR-223 on macrophage apoptosis in response to *Mtb* infection [27]. The essential role of FOXO3 in the regulation of anti-TB-directed immune responses may be one of the reasons why TB patients with hypomethylated *FOXO3* at the gene body had a higher all-cause mortality rate one year after the enrollment in this study. G protein subunit gamma 12 (GNG12) is a novel negative regulator of LPS-induced inflammation by down-regulating nitrite and TNF-α levels [28]. We speculate that hypermethylated *GNG12* epigenotype may predispose subjects to infection with *Mtb* and poorer outcomes by dampening host immune responses.

There are three autophagy-related genes (ATG) identified and verified in this study, while ATG signaling is the most enriched pathway associated with active TB disease. MiR505-ATG12 has been shown to be a vital signaling cascade for axonal development via inhibiting the autophagy pathway [29]. Mir-505 is also implicated in the repair strategies in influenza-induced murine pulmonary injury, and in the repression of porcine reproductive and respiratory syndrome virus [30,31]. RPTOR is a regulatory associated protein of mechanistic target of rapamycin, and involved in initiating T follicular regulatory cell differentiation by activating the TCF-1-Bcl-6 axis during immunization or infection [32]. It may be degraded by the periodontal pathogen Porphyromonas gingivalis during its invasion [33]. Mammalian ATG18 (WIPI2) is required for the formation of LC3-positive autophagosomes, and can promote macrophage migration and anti-bacterial autophagy [34,35]. ATGs have been shown to play a unique role in protection against *Mtb* by preventing neutrophil-mediated immunopathology [36]. Our results provide further evidence that altered DNA methylation of the *miR505*, *RPTOR*, and *WIPI2* genes may underlie autophagy-mediated immune responses to *Mtb*. Verification and validation of many enriched pathways identified in the discovery phase are ongoing. Finally, mitochondrial ribosomal protein S18B (MRPS18B) has an essential function in cardiac mitochondrial homeostasis [37]. Underlying mechanisms by which the altered DNA methylation levels of the candidate genes affect clinical phenotypes or outcomes of active TB disease remain to be investigated.

Several limitations in this study should be addressed. First, aberrant DNA methylation changes of many candidate genes were not accompanied by corresponding gene expression changes in both clinical PBMC samples and THP-1 cell lines exposed to *Mtb*-specific antigens, suggesting that the aberrant DNA methylation patterns did not directly invoke transcriptional modulation upon infection. Likewise, a recent genome-wide study on dendritic cells showed that virtually all changes in gene expression in response to *Mtb* infection and the binding of most infection-induced transcription factors occur prior to detectable, persisted alterations in the methylome [17]. Thus, aberrant DNA methylation in response to *Mtb* infection could have a specific biological role in trained innate immune memory rather than regulate immune gene expressions upon infection. Second, the sample size of the discovery cohort used for the whole-genome microarray experiment was relatively small. However, several DNA methylation alteration patterns identified in the microarray analysis were validated in another independent cohort by pyrosequencing method. Finally, the cause and effect relationships between aberrant DNA methylation patterns and active TB were not straightforward in this association study. However, the intersection of comparison I and II showed that only a few aberrant DNA methylation patterns were reversed after anti-TB treatment, and the preliminary in vitro experiments showed that similar methylation changes could be induced by ESAT6 or CFP10 in only 2 of the 9 selected genes, suggesting that most of the aberrant DNA methylation patterns might be present before the infection of *Mtb* and contribute to the susceptibility to active TB disease, namely inheriting epigenotypes. It is also possible that the aberrant methylation patterns occurred after chronic *Mtb* infection or reactivation and persisted after clearance of the infection, contributing to the faster and stronger transcriptional response observed upon re-stimulation (trained innate immunity).

## 4. Materials and Methods

### 4.1. Study Subjects

This study was approved by the Institutional Review Board of Chang Gung Memorial Hospital, Taiwan (certificate number: 103-7025B). The study participants were recruited from the Pulmonary Department of Kaohsiung Chang Gung Memorial Hospital from January 2015 through July 2018. Written informed consent was obtained from each subject participating in the study. The criteria for enrollment of disease group were clinical and radiological findings indicating pulmonary TB, and at least 1 positive *Mtb* culture from sputum or bronchial washing specimen obtained by bronchoscopy. Patients with HIV, malignancy, or concomitant infection other than *Mtb* were excluded. Acid fast bacilli (AFB) smears were performed, and standard postero-anterior chest X-rays (CXR) were assessed [38]. The criteria for enrollment of control group were the absence of pulmonary lesions on CXR examination and a negative history of TB disease. The discovery cohort used for the whole-genome DNA methylation microarray experiment included 12 newly diagnosed pulmonary TB patients, 3 patients after 6-month anti-TB therapy, and 6 healthy subjects. The independent validation cohort included 64 active TB patients at diagnosis, 18 TB patients after 6-month anti-TB therapy, and 24 HS. All patients were treated in accordance to the Taiwan and American Thoracic and Infectious Society guidelines for the management of TB, and received directly observed treatment, short course strategy [39].

### 4.2. Genome-Wide DNA Methylation Assay and Data Analysis

PBMCs were isolated from heparinized blood of all study subjects by Histopaque 1.077 (Sigma Diagnostics, St.Louis, MO, USA), and DNA was extracted using Puregene Core kit (Qiagen, Maryland, USA). Infinium HumanMethylation450K BeadChip v1.2 (San Diego, CA, USA) was used to detect 482,421 methylation CpG sites of 14,495 genes. The Methylation Module in the Illumina Genome Studio V2009.2 (San Diego, CA, USA) was used to generate the methylation intensity (β value) for each CpG locus, which was then transformed into a M-value to achieve better statistical properties [40]. To identify differential methylated CpG sites, M values of the case and control groups were analyzed with the Mann-Whitney test by Partek ^®^ Genomics Suite ^®^ (Missouri, USA) software, with the significance threshold of <0.0005 for a *p* value and <0.5 for a false discovery rate (q). Significantly differentially methylated CpG sites with at least a 10% difference in their β value (large effect size) and known biological or functional relevance, were selected for further validation [41]. For the differentially methylated CpG sites, their corresponding gene symbols were used for pathway analysis using MetaCore from Thomson Reuters (Philadelphia, USA). All methylation datasets have been deposited in the NCBI Gene Expression Omnibus with the accession number GSE118469. Additional detail on the method for making these measurements and analyses is provided in supplementary material in an online data supplement.

### 4.3. Measurement of DNA Methylation and Gene Expression Levels of Selected Genes in the PBMC Samples from the Validation COHORT

Genomic DNA (0.5 μg) was bisulphite-modified using the EZ DNA methylation kit (Zymo Research Corp, Orange County, CA). Polymerase chain reaction (PCR) was carried out with biotinylated forward primers and reverse primers for 21 selected genes (supplementary Table S1), using the following PCR program: 95 °C for 5 min, then 45 cycles of 95 °C for 30 sec followed by 56~61 °C for 30 sec and 72 °C for 30 sec, with a final extension at 72 °C for 5 min. Pyrosequencing was conducted using the PyroMark Q24 1.010 instrument and software (Qiagen). The amount of C relative to the sum of the amounts of C and T at each CpG site was calculated as the percentage of methylated cytosines [6].

Pyro-sequencing was applied to validate the DNA methylation levels over 26 CpG sites of 21 selected genes identified by the microarray experiment, including *RNASE3, MRPS18B, LGALS3, MIR223, ICAM2, GHRL, MIR505, PARP9, PLCL2, WIPI2, PYCR2, ITSN1, ICOS, FOXO3, RPTOR, CCR6, CASP8, GNG12, RASGRP4, GZMK*, and *MAP1LC3* (Table S4). Primer sequences used in quantitative RT-PCR for the 9 candidate genes are as listed in Table S5.

### 4.4. In Vitro Cell Culture Model under Specific TB Antigen Stimuli

Human THP-1 monocytic cells (1 × 106 cells) were seeded into a 96-well plate for 24 h, and stimulated with pre-specified concentrations of the recombinant proteins for 48 h: 1/10 μg/mL for 6 kDa early secretory antigenic target (ESAT-6), 5/10 μg/mL for 10 kDa culture filtrate antigenic protein (CFP-10). All experiments were performed in quadruplicate, independently. Lipopolysaccharide (LPS) was used as positive control. Wells left un-stimulated were negative control. DNA methylation levels and gene *expressions were measured as described above.*

### 4.5. Statistical Analysis

Continuous values were expressed as mean ± standard deviation. The differences between two groups were analyzed using the independent Student’s t-test or X2-test, as appropriate. The differences of continuous variables among more than two groups were analyzed using the ANOVA test. Multivariate linear regression analysis was performed to adjust for all potential confounding factors, including age, sex, smoking history, alcoholism, and co-morbidities. The Kaplan–Meier method was used to evaluate epigenetic biomarkers associated with one-year overall survival. The null hypothesis was rejected at *p* < 0.05. All analyses were performed using SPSS software version 19.0 (SPSS Corp., Chicago).

## 5. Conclusions

In summary, we reported a novel association of active pulmonary TB disease in adults of Asian origin with aberrant DNA methylation in the gene promoter or enhancer regions of the *miR-505*, *PARP9*, *RASGRP4*, and *GNG12* genes in blood immune cells. The findings extend reports linking *PARP9*/*miR-505*/*MRPS18B*/*RPTOR*/*FOXO3* hypomethylation and *RASGRP4*/*WIPI2*/*GNG12* hypermethylation with the clinical phenotypes, or survival in active TB patients, and provide direct evidence that perturbation of *PARP9*/*RASGRP4*/*WIPI2*/*FOXO3* signaling through epigenetic programming may play an important role in the mediation of human immune responses against *Mtb*. In conclusions, we present whole genome DNA methylation analysis in patients with active pulmonary TB and validated several differentially DNA methylation loci by pyro-sequencing in an independent cohort. Aberrant DNA methylation patterns of the *PARP9*, *miR-505*, *RASGRP4*, and *GNG12* showed the most prominent association with active TB disease, while aberrant DNA methylation patterns of the *GNG12*, *WIPI2*, *MRPS18B*, and *FOXO3* genes could predict one-year all-cause mortality. These findings may serve as a founding for developing new epigenetic targets for early diagnosis or host-directed therapy in TB.

## Figures and Tables

**Table 1:** Demographic and clinical characteristics in the sputum culture positive pulmonary tuberculosis (TB) patients and healthy subjects in the discovery and validation cohorts.

Characteristics	Discovery Cohort			Validation Cohort		
	TB patients *N* = 12	Healthy Subjects *N* = 6	*p* Value *	TB Patients *N* = 64	Healthy Subjects *N* = 24	*p* Value *
Age, years	65.8 ± 12.1	64.3 ± 4.7	0.775	62.7 ± 14.3	62.9 ± 8.4	0.939
Male sex, n (%)	12 (100)	6 (100)	0.587	46 (71.9)	17 (70.8)	0.923
Co-morbidity, n (%)						
Hypertension	2 (16.7)	0 (0)	0.289	12 (18.8)	3 (12.5)	0.487
Diabetes mellitus	4 (33.3)	1 (16.7)	0.457	22 (34.4)	7 (29.2)	0.643
COPD/Asthma	3 (25)	0 (0)	0.18	7 (10.9)	4 (16.7)	0.469
Chronic hepatitis	1 (8.3)	2 (33.3)	0.187	12 (18.8)	3 (12.5)	0.487
Chronic kidney disease	3 (25)	0 (0)	0.18	8 (12.5)	1 (4.2)	0.251
Heart failure	1 (8.3)	0 (0)	0.467	5 (7.8)	3 (12.5)	0.496
Alcoholism, n (%)	1 (8.3)	0 (0)	0.467	5 (7.8)	1 (4.2)	0.546
Smoking history, n (%)	4 (33.3)	1 (16.7)	0.457	19(29.7)	6 (25)	0.664
Sputum smear at diagnosis, n (%)						
Acid fast bacilli 0	1 (8.3)			16 (25)		
Acid fast bacilli 1+	1 (8.3)			14 (21.9)		
Acid fast bacilli 2+	0 (0)			10 (15.6)		
Acid fast bacilli 3+	6 (50)			10 (15.6)		
Acid fast bacilli 4+	4 (33.3)			14 (21.9)		
Drug-resistant TB, n (%)	4 (33.3)			11 (17.2)		
CXR at diagnosis, n (%)						
Far advanced lesions	9 (75)			38 (59.4)		
Minimal to moderate	3 (25)			26 (40.6)		
Systemic symptoms, n (%)	21 (25.9)			13 (23.6)		
Fever	9 (11.1)			3 (5.5)		
Body weight loss	2 (12.7)			6 (10.9)		

* Comparisons between TB patients and healthy subjects by independent t-test or chi-square test as indicated.

**Table 2:** Top differentially methylated loci (DMLs) in the comparison between active TB patients and healthy subjects (comparison I).

UCSC RefGene Name	UCSC RefGene Accession	UCSCRefGene Group	Chromosome	Mean Difference	*p*-Value	q Value	Column ID
*PLEKHG4B*	NM_052909	Body	5	−0.31	0.000101145	0.443008	cg18816122
*LARP4B*	NM_015155	Body	10	−0.251	0.000571043	0.221504	cg05931497
*KRT17*	NM_000422	TSS1500	17	−0.237	0.000259746	0.221504	cg08058191
*KIAA1257*	NM_020741	Body	3	−0.223	0.00731796	0.443008	cg25752703
*ETF1*	NM_004730	3’UTR	5	−0.213	0.00201464	0.443008	cg21141827
*LRPAP1*	NM_002337	Body	4	−0.211	0.00277208	0.443008	cg08472008
*AATK*	NM_001080395	Body	17	−0.207	0.00167958	0.443008	cg20814202
*ELL2*	NM_012081	Body	5	−0.206	0.00299245	0.443008	cg16998950
*ITSN1*	NM_003024	5’UTR	21	−0.205	0.00352359	0.443008	cg16452651
*LOC100270710*	NR_026754	TSS200	10	−0.2	0.000444703	0.221504	cg27570256
*ADAP1*	NM_006869	Body	7	−0.196	4.12 × 10^−5^	0.110752	cg18332229
*KCNQ1*	NM_000218	Body	11	−0.195	0.00712947	0.110752	cg03155200
*FBXO6;FBXO44*	NM_018438	TSS1500;3’UTR	1	−0.194	0.00438873	0.443008	cg01554529
*HDAC4*	NM_006037	Body	2	−0.194	0.00271246	0.110752	cg22077197
*KIFC1*	NM_002263	TSS1500	6	−0.183	0.000102932	0.110752	cg15206171
*ARNT*	NM_178427	Body	1	−0.181	3.82 × 10^−5^	0.221504	cg00944785
*AUH*	NM_001698	Body	9	−0.181	0.000697739	0.443008	cg13932501
*MTHFR*	NM_005957	Body	1	−0.181	0.000697402	0.221504	cg17514528
*FLJ22536*	NR_015410	Body	6	−0.178	0.00158527	0.221504	cg16804603
*PRKCZ*	NM_001033581	Body	1	−0.176	0.0015181	0.443008	cg17435831
*ZNF827*	NM_178835	Body	4	−0.176	0.00195335	0.443008	cg19116959
*SBNO2*	NM_014963	5’UTR	19	−0.175	0.00141277	0.443008	cg18004847
*NID1*	NM_002508	Body	1	−0.174	0.00382673	0.443008	cg26837399
*BCOR*	NM_001123384	5’UTR	X	−0.173	0.00514341	0.443008	cg10055320
*IPCEF1;OPRM1*	NM_001130699	Body	6	−0.173	0.000163433	0.110752	cg27296341
*CHMP4B*	NM_176812	TSS1500	20	−0.172	0.000977403	0.443008	cg03355213
*NDUFS2*	NM_004550	5’UTR	1	−0.172	0.00099	0.221504	cg05656486
*TEAD3*	NM_003214	Body	6	−0.172	0.00240499	0.443008	cg23254569
*GFI1*	NM_005263	TSS1500	1	−0.171	0.0036827	0.443008	cg09674502
*WDFY4*	NM_020945	TSS200	10	−0.17	0.000167422	0.110752	cg04749316
*ATP5A1;HAUS1*	NM_001001937	5’UTR;TSS1500	18	−0.168	0.00584364	0.221504	cg18069568
*PTPN7*	NM_002832	Body	1	−0.168	0.00219543	0.443008	cg17932662
*SRBD1*	NM_018079	Body	2	−0.165	0.00426648	0.443008	cg12939390
*BRF1*	NM_001519	Body	14	−0.164	0.00796682	0.443008	cg19676553
*C21orf56*	NM_032261	5’UTR; 1stExon	21	−0.164	0.000173268	0.221504	cg25545878
*KLF5*	NM_001730	Body	13	−0.164	0.009382	0.110752	cg04339360
*GSN*	NM_001127664;	5’UTR	9	−0.163	0.00313606	0.443008	cg13569051
*RBM47*	NM_001098634	5’UTR	4	−0.163	0.00304298	0.110752	cg20789700
*TBCD*	NM_005993	Body	17	−0.163	0.0172711	0.443008	cg06218079
*TLR4*	NM_138554	Body;3’UTR	9	−0.161	0.00184428	0.443008	cg14605396
*ANKRD34B*	NM_001004441	5’UTR	5	−0.158	0.00012512	0.110752	cg24834873
*DEPDC1*	NM_001114120	TSS1500	1	−0.158	0.000861252	0.443008	cg18167921
*CLCC1*	NM_001048210	Body	1	−0.157	0.00119716	0.443008	cg06756164
*CUBN*	NM_001081	Body	10	−0.157	0.00110262	0.110752	cg07732336
*CSGALNACT1*	NM_018371	TSS200	8	−0.156	0.00218679	0.443008	cg18325192
*C19orf71*	NM_001135580	TSS1500	19	−0.155	0.0232128	0.443008	cg17344770
*EDARADD*	NM_080738	Body	1	−0.155	0.0455698	0.443008	cg15086439
*MGAT5B*	NM_144677	TSS1500	17	−0.155	0.000162922	0.443008	cg25923214
*SLFN12L*	NM_001145027	Body	17	−0.155	0.000474321	0.443008	cg11855615
*SSH1*	NM_001161330	Body	12	−0.155	0.00924305	0.221504	cg21224380
*WBSCR17*	NM_022479	Body	7	−0.153	2.51E-05	0.221504	cg16533336
*HOXB2*	NM_002145	TSS200	17	−0.152	0.000734653	0.221504	cg09313705
*SPRYD4*	NM_207344	3’UTR	12	−0.152	0.0067393	0.110752	cg17554464
*INF2*	NM_022489	5’UTR	14	−0.149	0.00905403	0.443008	cg01836137
*MIR223*	NR_029637	TSS200	X	−0.149	0.000317282	0.110752	cg19127840
*RNASE3*	NM_002935	5’UTR	14	−0.149	0.000488913	0.221504	cg09842118
*AOAH*	NM_001637	Body	7	−0.148	0.00129482	0.221504	cg00390511
*PARP9;DTX3L*	NM_001146106	5’UTR; TSS1500	3	−0.148	0.00594526	0.110752	cg22930808
*MRPS18B;PPP1R10*	NM_014046;NM_002714	Body;TSS1500	6	0.113	0.000298705	0.221504	cg04176995
*ABCC1*	NM_019862	Body	16	0.116	1.33 × 10^−5^	0.110752	cg04981696
*ERH; SLC39A9*	NM_004450	1stExon;5’UTR;TSS1500	14	0.116	5.09 × 10^−5^	0.221504	cg04509559
*BPI*	NM_001725	TSS200	20	0.117	0.000144623	0.443008	cg01948217
*EIF2C2*	NM_012154	Body	8	0.123	0.00544777	0.443008	cg19352830
*PLAG1;CHCHD7*	NM_001114635	5’UTR;TSS1500;	8	0.124	2.14 × 10^−5^	0.110752	cg01994308
*LOC400759*	NR_003133	TSS1500	1	0.138	3.85 × 10^−6^	0.110752	cg13454346
*HSPA8*	NM_006597	Body	11	0.139	0.00331157	0.221504	cg03309938
*LIME1*	NM_017806	TSS1500	20	0.139	2.92 × 10^−6^	0.110752	cg14977069
*CPT1A*	NM_001876;NM_001031847	5’UTR;5’UTR	11	0.152	7.96 × 10^−6^	0.110752	cg19081843
*RPTOR*	NM_001163034	Body	17	0.164	0.000764449	0.443008	cg10035831
*MICAL3*	NM_015241	TSS200	22	0.18	1.51 × 10^−5^	0.000394	cg05367846

NCBI=National Center for Biotechnology Information; TSS= transcription start site; UTR= un-translated region.

## Supplementary Materials

Supplementary materials can be found at https://www.mdpi.com/1422-0067/21/9/3180/s1.

## Abbreviations

Mtb	mycobacterium tuberculosis
CpG	cytosine guanine di-nucleotides
PBMC	peripheral blood mononuclear cell
LD	linear dichroism
HS	healthy subject
AFB	acid fast bacilli
CXR	chest X-radiograph
PCR	polymerase chain reaction
ESAT-6	early secretory antigen target 6
CFP-10	culture filtrate antigenic protein 10
LPS	lipopolysaccharide
DML	differentially methylated loci
