# Supplementary material for: Whole Genome DNA Methylation Analysis of Active Pulmonary Tuberculosis Disease Identifies Novel Epigenotypes: PARP9/miR-505/RASGRP4/GNG12 Gene Methylation and Clinical Phenotypes
Source: Int J Mol Sci. 2020 Apr 30;21(9):3180. doi: 10.3390/ijms21093180 (PMC7246806; doi:10.3390/ijms21093180)
Supplement: Supplementary file 1 [file ijms-21-03180-s001.pdf]

Whole Genome DNA Methylation Analysis of Active Pulmonary Tuberculosis Disease Identifies Novel Epigenotypes: *parp9/mir-505/rasgrp4/gng12* Gene Methylation and Clinical Phenotypes

Yung-Che Chen[1, 6, #], Chang-Chun Hsiao[1, 5], Ting-Wen Chen[7, 8], Chao-Chien Wu[1], Tung-Ying Chao[1], Sum-Yee Leung[1], Chiu-Ping Lee[1], Hock-Liew Eng[2], Chien-Hung Chin[1], Chia-Cheng Tseng[1], Huang-Chih Chang[1], Wen-Feng Fang[1], Chung-Jen Chen[3], Ting-Ya Wang[1], Yong-Yong Lin[1], Jen-Chieh Chang[4], Meng-Chih Lin[1, #]

Supplementary text

Isolation of DNA, RNA, and protein from PBMC samples

PBMCs were isolated from heparinized blood of all study subjects using a Ficoll-Histopaque density gradient centrifugation (Histopaque 1.077 and 1.119; Sigma Diagnostics, St.Louis, MO, USA) method. Blood samples at diagnosis were obtained and analyzed from all TB patients and HS, and after six months of anti-TB treatment from selected patients. Samples were stored in RNA*later*® RNA stabilization solution (Ambion®) at -80 ℃ until analysis. DNA was extracted using Puregene Core kit (Qiagen, Maryland, USA). An RNeasy® Plus Mini Kit (Qiagen, Hilden, Germany) was used for isolation of RNA, and treated with DNase according to the manufacturer's protocol.

Genome-wide DNA methylation assay

Infinium HumanMethylation450K BeadChip v1.2 (San Diego, CA, USA) was used to detect 482,421 methylation CpG sites of 14,495 genes, with the distance to the transcription start site ranging from 0 to 1499 bp. Electropherograms using an Agilent BioAnalyzer with Agilent DNA 12000 chips showed the fragment size to be >10000 bp. For bisulfite conversion, EZ DNA methylation kit (Zymo Research, USA) was used. About 200 ng of each bisulfite-converted genomic DNA sample was applied per BeadChip[1,2].

Genome-Wide DNA Methylation Data Analysis

The Methylation Module in the Illumina Genome Studio V2009.2 (San Diego, CA, USA) was used to generate the β value for each CpG locus. The β value was calculated as: (intensity of methylated probe)/(intensity of methylated probe + intensity of unmethylated probe). The β-values ranged between 0 (least methylated) and 1 (most methylated) and was then transformed into a M-value to achieve better statistical properties [3], which was the $\log_2$ ratio of the intensity of methylated probes versus unmethylated probes using the following equation: M value = $\log_2$ (β value/(1-β value)). A positive M-value meant higher intensity from the methylated probes than the un-methylated probes and a negative M value meant the opposite. The significance threshold in M value comparisons was $p < 0.005$ and a false discovery rate (q) <0.5.

To identify differential methylated CpG sites, M values of the case and control groups were analyzed with the Mann–Whitney test by Partek ® Genomics Suite ® software to obtain a p value and q value, as described previously[2]. Significantly differentially methylated CpG sites with a p value < 0.005, q value < 0.01, at least a 10% difference in their β value (large effect size), and known biological or functional relevance were selected for further validation[4]. For the differentially methylated CpG sites, their corresponding gene symbols were used for pathway analysis using MetaCore from Thomson Reuters, which uses hypergeometric tests to examine whether the genes are enriched in any known pathway. The top 10 pathways were selected based on their p values (<0.005) and q values (<0.25). All methylation datasets have been deposited in the NCBI Gene Expression Omnibus with the accession number GSE118469.

Measuring Candidate Gene Expressions of Peripheral Blood Mononuclear Cells by Quantitative Realtime Reverse Transcription (RT)-PCR Method

Total RNA from PBMCs was isolated by RNA Extraction RiboPureTM-Blood (Ambion), and converted to single stranded cDNA using a cDNA archive kit (Applied

Biosystems) followed by the amplification of specific gene transcript by using TaqMan probe and specific primers (supplementary Table S2). *GAPDH* was used as the internal control. The PCR reaction was performed at 94 ℃ for 10 min, followed by amplification (95 ℃ for 10 s, 60 ℃ for 30 s), and cooling (40 ℃ for 30 s), for 30 cycles. The PCR products were subjected to 1% agarose gel electrophoresis and photographed. Relative expression levels were calculated using the ΔΔCq method with the median value for the HS group as the calibrator. All amplification reactions were performed simultaneously.

Analysis of *miRNA-505* Gene Expression

cDNA was generated from 2 μL of purified total RNA using the TaqMan Advanced miRNA cDNA Synthesis kit (Thermo Fisher Scientific, Waltham, MA, United States). Additionally, 1 pM of the synthetic *C. Elegans oligo*, cel-miR-39 (Sequence: UCACCGGGUGUAAAUCAGCUUG), was added to the isolated total RNA. This sequence does not exist in humans and was used as an exogenous control. All qPCR reactions were normalized to their corresponding cel-miR-39 C_t values. Quantitative RT-PCR was performed for each sample using 2.5 μL of diluted cDNA, TaqMan Advanced miRNA Assays (cel-miR-39-3p: 478293_mir ; hsa-miR-505-5p: 478957_mir ; Thermo Fisher Scientific, Waltham, MA, United States), and Applied Biosystems™ TaqMan™ Fast Advanced Master Mix (Thermo Fisher Scientific, Waltham, MA, United States) under fast cycling conditions. All TaqMan assays quantitative RT-PCR was carried out using the ABI 7500fast Real-Time PCR System (Applied Biosystems). Real-time PCR cycling conditions consisted of 95 ℃ for 20 s, followed by 40 cycles of 95 ℃ for 3 s and 60 ℃ for 30 s. miRNA fold expression changes were determined by the $2^{-\Delta\Delta CT}$ method.

References

1 Bibikova, M. *et al.* Genome-wide DNA methylation profiling using Infinium(R) assay. *Epigenomics* **1**, 177-200, doi:10.2217/epi.09.14 (2009).

2 Chen, Y. C. *et al.* Whole Genome DNA Methylation Analysis of Obstructive Sleep Apnea: IL1R2, NPR2, AR, SP140 Methylation and Clinical Phenotype. *Sleep* **39**, 743-755, doi:10.5665/sleep.5620 (2016).

3 Du, P. *et al.* Comparison of Beta-value and M-value methods for quantifying methylation levels by microarray analysis. *BMC bioinformatics* **11**, 587, doi:10.1186/1471-2105-11-587 (2010).

4 Michels, K. B. *et al.* Recommendations for the design and analysis of epigenome-wide association studies. *Nature methods* **10**, 949-955, doi:10.1038/nmeth.2632 (2013).

Table S1. Top differentially methylation loci (DML) in the comparison before and after anti-TB treatment (comparison II).

NCBI RefGene Name	NCBI RefGene Accession	NCBI RefGene Group	Chromosome	Mean difference	p-value	q-value	Column ID
RPL9; LIAS	NM_001024921	5'UTR;TSS200	4	0.364	0.125497	0.004396	cg19311470
TRNT1	NM_182916	Body		0.312	0.000132626	0.004396	ch.3.55501R
COG5	NM_006348	Body	7	0.244	0.0731974	0.004396	cg23024343
HCG4P6	NR_001317	Body	6	0.2	0.00888886	0.004396	cg04246123
GLT25D2	NM_015101	TSS1500	1	0.185	0.00977335	0.004396	cg01068808
PIAS3	NM_006099	TSS1500	1	0.182	8.17E-07	0.004396	cg04921814

NCEH1	NM_001146278	5'UTR;Body	3	0.182	5.07E-05	0.004396	cg10532262
SLC25A33	NM_032315	Body	1	0.16	0.0113401	0.004396	cg26607031
SVIL;SVIL	NM_021738;	TSS1500;5'UTR		0.148	0.0332648	0.004396	cg11316887
ITPR1;EGOT	NM_002222	Body; TSS1500		0.146	0.0705987	0.004396	cg11600734
DACH2	NM_001139514	TSS1500	X	0.145	0.00590437	0.004396	cg25443545
VWC2	NM_198570	1stExon; 5'UTR	7	0.144	9.70E-05	0.004396	cg18206027
EVX2	NM_001080458	TSS200	2	0.136	0.0010306	0.004396	cg15133351
WRNIP1	NM_130395	Body		0.135	0.00677545	0.004396	cg13598881
PCDHA2	NM_018905	Body; TSS200	5	0.132	0.000449357	0.004396	cg09820378
F2RL1	NM_005242	Body	5	0.131	0.0261704	0.004396	cg18586277
HDAC4	NM_006037	5'UTR		0.127	0.0159904	0.004396	cg02708956
CASZ1	NM_001079843	3'UTR	1	0.126	0.0145848	0.004396	cg16101008
LIME1	NM_017806	TSS200	20	0.126	0.00279738	0.004396	cg06653796
TRIM15	NM_033229	3'UTR	6	0.124	0.0104508	0.004396	cg03771840
NOS1AP	NM_014697	Body		0.121	0.00164369	0.004396	cg02455571
HS3ST1	NM_005114	TSS200	4	0.12	0.00028692	0.004396	cg01756381
PTPRG	NM_002841	Body		0.118	0.0244711	0.004396	cg11934688
IRX2; C5orf38	NM_033267	TSS1500;TSS200	5	0.117	0.000645891	0.004396	cg21093166
FAIM3	NM_001142473;	Body	1	0.116	0.0033914	0.004396	cg21607172
LSM11	NM_173491	Body	5	0.116	0.000996015	0.004396	cg14126863
PRDM16	NM_022114	Body	1	0.114	0.0424537	0.004396	cg11731671
C1orf70	NM_001114748	Body	1	0.113	0.00103542	0.004396	cg08738570
GNG12	NM_018841	5'UTR	1	0.113	0.00196249	0.004396	cg17931620
CCR6	NM_004367	5'UTR;TSS200		-0.123	0.00731069	0.004396	cg21794222
RASGRP4	NM_001146202;	TSS1500		-0.123	0.0120585	0.004396	cg24376214
C5orf20	NM_1308	TSS1500		-0.126	0.00175	0.00	cg1357

	48				248	4396	7149
PCDHA7	NM_018910	Body	5	-0.126	0.00128697	0.004396	cg24915503
SCGN	NM_006998	Body	6	-0.126	0.00781617	0.004396	cg03349134
REG4	NM_001159352	Body		-0.127	0.0035239	0.004396	cg19810433
WNT16	NM_016087	TSS1500		-0.129	9.64E-05	0.004396	cg24849648
ATP10B	NM_025153	5'UTR		-0.13	0.00409421	0.004396	cg24639117
ZNF311	NM_001010877	Body		-0.13	0.00086073	0.004396	cg26380692
EXOC2	NM_018303	Body	6	-0.131	0.0101604	0.004396	cg27562005
PRSS35	NM_153362	5'UTR		-0.131	0.00189682	0.004396	cg16162611
LOC100129534;MORN1	NR_024489	Body		-0.132	6.58E-05	0.004396	cg13805052
RAPGEF3	NM_001098532	Body		-0.133	0.107617	0.004396	cg23815853
HHATL	NM_020707	5'UTR;1stExon;TSS1500		-0.135	0.00179958	0.004396	cg19827650
MBNL3	NM_133486	Body;TSS200;5'UTR		-0.137	0.00858141	0.004396	cg14520512
PPP2R2B	NM_181677	5'UTR;TSS1500		-0.138	0.000334285	0.004396	cg08991927
CACNA1D	NM_001128839	Body		-0.139	0.000510919	0.004396	cg13757263
KCNQ1	NM_000218	Body		-0.14	0.037976	0.004396	cg17416793
SDK1	NR_027816	Body		-0.142	6.60E-05	0.004396	cg25783987
MIR548A2	NR_030317	Body		-0.143	0.00425148	0.004396	cg02917236
TBC1D9	NM_015130	Body		-0.145	0.000100421	0.004396	cg22631616
SGCD	NM_001128209;NM_172244;NM_000337	Body		-0.154	0.000187951	0.004396	cg26676094
MYOF	NM_133337	Body		-0.156	0.0676326	0.004396	cg11276093
LOC285768	NR_027116	Body		-0.157	0.00304634	0.004396	cg21823426
CHL1	NM_006614	5'UTR	3	-0.163	0.0210124	0.004396	cg08707471
VOPP1	NM_0307	Body	7	-0.174	0.01629	0.00	cg0688

	96				12	4396	9086
SST	NM_001048	TSS1500	3	-0.187	0.0129487	0.004396	cg14703224
ARID5A	NM_212481	Body	2	-0.189	0.027759	0.004396	cg04722215
SH3PXD2B	NM_001017995	Body		-0.191	0.00600959	0.004396	cg19979108
VANGL2	NM_020335	Body	1	-0.194	0.000346465	0.004396	cg06928484
RPS6KC1	NM_001136138	TSS200	1	-0.198	0.0185273	0.004396	cg14576824
11-Mar	NM_001102562	Body	5	-0.223	0.00108518	0.004396	cg03477332
GZMK	NM_002104	TSS200		-0.277	0.0321398	0.004396	cg03979311
MAST2	NM_015112	Body		-0.374	0.0483769	0.004396	cg22337626

Table S2. Top 10 enriched pathways in the comparison between TB patients and healthy subjects (comparison I).

Maps	In Data/ Total	P Value	Min FDR	Genes from Active Data
Autophagy_Autophagy	6/32	1.218E-04	0.06381071	*APG16L1, DAPK1, Raptor, Beclin 1, ULK1, Endophilin B1*
Regulation of metabolism_Bile acids regulation of glucose and lipid metabolism via FXR	6/37	2.814E-04	0.07372728	*GSK3 alpha/beta, SCD, FASN, HNF3-alpha, HNF3-beta, HNF3*
Cell adhesion_Ephrin signaling	6/45	8.344E-04	0.1237689	*Ephrin-A, Kalirin, Ephrin-A5, TAK1(MAP3K7), Intersectin, Ephrin-A2*
Apoptosis and survival_Role of IAP-proteins in apoptosis	5/31	9.448E-04	0.1237689	*HSP70, c-IAP1, c-IAP2, FasR(CD95), Aif*
Regulation of lipid metabolism_Regulation of lipid metabolism via LXR, NF-Y and	5/38	2.421E-03	0.2418127	*AMPK gamma subunit, SCD, FASN, YY1, Caveolin-1*

SREBP				
DNA damage_Role of NFBD1 in DNA damage response	3/13	3.694E-03	0.2418127	*ATR, Chk2, p53BP1*
Regulation of degradation of deltaF508 CFTR in CF	4/27	4.291E-03	0.2418127	*HSP70, UBE2D1, VCP, HSC70*
Apoptosis and survival_Anti-apoptotic TNFs/NF-kB/IAP pathway	4/27	4.291E-03	0.2418127	*I-kB, c-IAP1, c-IAP2, CD30(TNFRSF8)*
CFTR folding and maturation (norm and CF)	3/14	4.615E-03	0.2418127	*HSP70, HSP40, UGCGL1*
Transport_RAB3 regulation pathway	3/14	4.615E-03	0.2418127	*Rab-3, Rab-3A, RAB3IP*

Table S3. Top 10 enriched pathways in the comparison before and after anti-TB treatment (comparison II).

Maps	In Data/Total	pValue	Min FDR	Genes from Active Data
Signal transduction_Activation of PKC via G-Protein coupled receptor	21/52	6.140E-10	4.2307E-07	*Sequestosome 1(p62), PKC-beta, PKC-mu, NF-AT4(NFATC3), GSK3 beta, PLC-beta, PKC-alpha, PKC-delta, ERK1/2, MEK2(MAP2K2), c-Abl, IKK-gamma, G-protein beta/gamma, MEF2, NF-kB, CPI-17, G-protein alpha-q/11, MELC, PKC-epsilon, NF-AT2(NFATC1), IP3 receptor*
Cardiac Hypertrophy_NF-AT signaling in Cardiac Hypertrophy	23/65	2.056E-09	7.0845E-07	*Shc, CBP, PKC-mu, ERK5 (MAPK7), GSK3 beta, PI3K reg class IA, GAB1, PKC-alpha, NCX1, G-protein alpha-i family, G-protein beta/gamma, MEF2A, HDAC4, ADSSL1, Cardiac MyBP-C, HDAC5, Beta-1 adrenergic receptor, AKT(PKB), G-protein alpha-q/11, Angiotensin II, PKC-epsilon, Troponin I, cardiac, alpha-MHC*
Neurophysiological process_ACM regulation of nerve impulse	18/46	1.899E-08	4.3618E-06	*RhoA, CACNA1H, TRPC3, PKC, PLC-beta, PKC-alpha, G-protein alpha-i family, G-protein alpha-o, G-protein alpha-11, L-type*

				Ca(II) channel, alpha 1C subunit, G-protein beta/gamma, FKBP12, G-protein alpha-q, PKA-reg (cAMP-dependent), G-protein alpha-q/11, CACNA1I, G-protein alpha-i2, IP3 receptor
Development_Gastrin in cell growth and proliferation	21/62	2.535E-08	4.366E-06	*PKC-beta, Shc, PKC-mu, PI3K reg class IA (p85), PI3K reg class IA (p85-alpha), MEKK1(MAP3K1), PKC-alpha, PKC-delta, TCF7L2 (TCF4), ERK1/2, MEK2(MAP2K2), G-protein alpha-q, Cyclin D1, JNK(MAPK8-10), ERK2 (MAPK1), G-protein alpha-q/11, Stromelysin-1, PKC-epsilon, ERK1 (MAPK3), p90Rsk, IP3 receptor*
Development_Ligand-independent activation of ESR1 and ESR2	16/45	5.553E-07	7.374E-05	*Shc, CBP, ErbB2, Galpha(s)-specific amine GPCRs, PI3K reg class IA, NCOA3 (pCIP/SRC3), Neuregulin 1, ERK1/2, MEK2(MAP2K2), ErbB3, Cyclin D1, PKA-reg (cAMP-dependent), NCOA1 (SRC1), ERK2 (MAPK1), AKT(PKB), ERK1 (MAPK3)*
Cytoskeleton remodeling_TGF, WNT and cytoskeletal remodeling	27/111	6.421E-07	7.374E-05	*Talin, RhoA, Shc, TGF-beta 1, GSK3 beta, PI3K reg class IA, Tcf(Lef), WNT, TGF-beta receptor type II, Destrin, TCF7L2 (TCF4), ERK1/2, MEK2(MAP2K2), LRP5, Cyclin D1, Alpha-actinin, SMAD3, ERK2 (MAPK1), Alpha-actinin 1, AKT(PKB), Collagen IV, MELC, LIMK1, p53, ERK1 (MAPK3), Axin, Frizzled*
Immune response_Gastrin in inflammatory response	20/69	9.601E-07	9.4501E-05	*ELAVL1 (HuR), RhoA, Shc, PI3K reg class IA (p85), ERK5 (MAPK7), MEKK1(MAP3K1), PKC-alpha, PKC-delta, MEK2(MAP2K2), G-protein alpha-q,*

				JNK(MAPK8-10), MEF2, ERK2 (MAPK1), AKT(PKB), NIK(MAP3K14), G-protein alpha-q/11, Stromelysin-1, PKC-epsilon, ERK1 (MAPK3), IP3 receptor
Development_Endothelin-1/EDNRA signaling	17/53	1.313E-06	0.0001131	*Shc, GSK3 beta, PI3K reg class IA, MEKK1(MAP3K1), PKC-delta, G-protein alpha-i family, Adenylate cyclase, TCF7L2 (TCF4), ERK1/2, MEK2(MAP2K2), Cyclin D1, JNK(MAPK8-10), AKT(PKB), G-protein alpha-q/11, PKC-epsilon, IP3 receptor, MEKK4(MAP3K4)*
Development_Thyroliberin signaling	18/60	1.929E-06	0.00014199	*RGS4, PKC-beta, Shc, CBP, PKC-alpha, PKC-delta, Adenylate cyclase, ERK1/2, MEK2(MAP2K2), G-protein alpha-11, L-type Ca(II) channel, alpha 1C subunit, G-protein beta/gamma, G-protein alpha-q, PKA-reg (cAMP-dependent), G-protein alpha-q/11, PKC-epsilon, G-protein alpha-i2, IP3 receptor*
Development_A3 receptor signaling	16/49	2.061E-06	0.00014199	*Shc, GSK3 beta, PLC-beta, G-protein alpha-i family, TCF7L2 (TCF4), MEK2(MAP2K2), G-protein beta/gamma, Cyclin D1, PKA-reg (cAMP-dependent), RASGRF1, NF-kB, AKT(PKB), G-protein alpha-q/11, PKC-epsilon, ERK1 (MAPK3), IP3 receptor*

Table S4. **Primer sequences used for PCR and pyro-sequencing of the 21 selected genes assayed in the validation cohort.**

Gene name /probe ID	CpG site location relative to transcription start site/ NCBI reference number	Primer Sequence 5'→3'
RNASE3 cg09842118	+175, NM_002935,	F.P:AGTGTTTAGGAGATGTGGTATAT<br>R.P: Biotin-AACCCACAAACCCCTCTAC<br>S.P:GGGGATAGGAAGAAAAG
MRPS18B cg04176995	+982, NM_014046,	F.P:AAGAGTGGTGATAGAGGTTAATATAGA<br>R.P: Biotin-AATCAATCCAATAAAATCACTAAATTACC<br>S.P:AAATAATAATAGTTTTGGTAGTT
MIR223 cg19127840	-95 NR_029637.1	F.P:AGAGAGAAAGGAATGATGAAGTTA<br>R.P: Biotin-ACTTCCCTATTCTAATACTTTAATTAATCC<br>S.P:GGAATGATGAAGTTATATTTTTAGT
LGALS3 cg04306507	-1321 NM_002306.3	F.P:AGGGAAATGTTTTTGTGAAGG<br>R.P: Biotin-CCCAACCACACTATAACTTCTATATACA<br>S.P:GGTTGGTAAGGTTTTTGTAATAT
ICAM2 cg12793803	+14263, NM_000873	F.P:TTTTTTGTATAGGGAGTTAGTAGGG<br>R.P: Biotin-ACTAATAACTACATTTCCTCTCATTATC<br>S.P:TTAGAGGTTTGGGGT
GHRL cg03751527	-243, NM_001134946,	F.P: Biotin-ATTGTATTTTAGTTTGGGTGATAGAG<br>R.P:AACATTACAACTTTAATCCCAAACCA<br>S.P:CCACAATAAAACAAATCACAC
ICOS cg18219180	-1120 NM_012092.3	F.P:ATGAGGAAAATTGAGGTATAGATAGG<br>R.P: Biotin-AAAATCCAACCTAAATCTAACATCTTAA<br>S.P:GTTTGTTGATTTTTTTATTTAGAAG
MIR505 cg16719099	-696, -690 NR_030230,	F.P:GGTTTGTTGATTTATTGGTAGAATTTAGT<br>R.P: Biotin-AACTTACACATAATTTCCTCCAAACTT<br>S.P:ATTGGTAGAATTTAGTATATAGAA

PARP9 cg22930808	+1741, NM_001146106,	F.P:AGGTTTTTGTATATGGTTGGTAAGAT<br>R.P: Biotin-CCCCCTATTTATAAACATTAAAAAATTCCC<br>S.P:AGATTGGAAATGGGT
PLCL2 cg20271057	+47700, NM_001144382,	F.P:GGAGAGGTTTTTTTTTTATAGTTATATGT<br>R.P: Biotin-ATTCATACCTTAAATCTATTTTCCCTACA<br>S.P:AGTTATATGTTGGTAGATAATG
ITSN1 cg16452651	+2167 NM_001331010.1	F.P: TGTTTGAGTAAGAGAGGAAATGAATAA<br>R.P: Biotin-CTTACTACCACTCATTTTATACCCTATTT<br>S.P: AGGAAATGAATAAATTTTTAATGTA
PYCR2 cg20334115	+4145, +4141 NM_013328,	F.P: Biotin-TTTAGTTTTTTTTTTTAGTTGGGTTGAT<br>R.P: CTCTCCTATCCCATATTAAAAATTACTC<br>S.P: ACCAAACACAACCCT
WIPI2 cg05639533	+28532, NM_016003	F.P: TTGAGGGGAGAGATGGTTT<br>R.P: Biotin-CTTATTCTCTTCCTCATTTAAACTATACC<br>S.P: GATTTTAAGATAGAAGAATG
FOXO3 cg06636172	+106809 NM_201559.2	F.P: GTTTTAGTTTTTTAAAGTGTTGGGATTATA<br>R.P: Biotin-AAAAATCCCAATATTCATCTTACTTCT<br>S.P: AGTGTTGGGATTATAGG
CCR6 cg15222091	+744 NM_004367.5	F.R: ATAGTTGAGATGTATGGAGAATTATT<br>R.P: Biotin-CTTTCAAAAAAAAAACCATAACAACTCTA<br>S.P: AGATGTATGGAGAATTATTT
CASP8 cg25073137	+26100 NM_001228.4	F.R: GAGGGTAGGGATTTATAGTTGAGA<br>R.P: Biotin-CCATCTTAACCTAACCCTCTCTAAT<br>S.P: GGATTTATAGTTGAGAAGATTG
GNG12 cg17931620	+1452 NM_018841.5	F.P: GTAAGGGTGTAGTTTGGTGATTAGTATT<br>R.P: Biotin-ACTTCCTTCCCATCAATTACT<br>S.P: GTTTTTAAAATAGAGAAGGTATAG
GZMK cg03979311	-111 NM_002104.2	F.P: TTATTATAGGTGTTTTAGGGGTAAGAT<br>R.P: Biotin-ACACAAACCTAAAAATCCCCAAACTA<br>S.P: AGGGGTAAGATTTTTTTTTATATT
MAP1LC3C cg14382888	+1307 XM_005273139.3	F.P: AGAAATTGGGTTTTTGGTTATATATAGG<br>R.P: Biotin-AAAAACACCTAACTAATATCCACTATAAA<br>S.P: GGGTGGGTGGGGGTT
RASGRP4 cg24376214	-1201; -1194; -1188 NM_001146202.1	F.P: GGAGGGTGAGAATGTTTTGT<br>R.P: Biotin-CCCATCCCCTACCTTTCTTAA<br>S.P: AGGAGTATATGTTTATATGATG
RPTOR		F.P: AGGGAAAGGTAGGAGGTTTTATA

cg10035831	+46451; +46448; +46392 NM_020761.2	R.P: Biotin-AAAACATCTACCAATAAAAATTTCACAT S.P: GTGATTATTGGGATTTTGG

Table S5. Primer sequences used for quantitative RT-PCR of the 9 candidate genes verified in the validation cohort.

Gene name		Primer sequence
PARP9	forward	5’- GGTTCTAAAGGTGGAGAAGATAGA-3’
	reverse	5’-GCATTGACACCTACCGCAA-3’
RASGRP4	forward	5’-CTGGTCAGGTACTGGCTGATG-3’
	reverse	5’-GCTGCTCATTGGGAGTGGG-3’
GNG12	forward	5’- ACACTCTTGGAATTTCCAGG -3’
	reverse	5’- AATGAACTGAAGAAGAATTAAAGCATC -3’
WIPI2	forward	5’-GCTCTTCGCCAACTTCAACC-3’
	reverse	5’-CCAACAGCTAGGGACCAGAC-3’
FOXO3	forward	5’-GCTCTTCGCCAACTTCAACC-3’
	reverse	5’-CCAACAGCTAGGGACCAGAC-3’
MRPS18B	forward	5’-GATATGGTTCTCGCCCCGTC-3’
	reverse	5’-CTCCAAGAGCTTCACGTTCCTA-3’
RPTOR	forward	5’-AAGATCCTCGCAGTGGACAG-3’
	reverse	5’-GGTGTTCAGCTGGCATGTAG-3’
CCR6	forward	5’- TTC AGC GAT GTT TTC GAC TCC-3’
	reverse	5’-GCA ATC GGT ACA AAT AGC CTG G-3’

Figure legends

Figure S1. Gene expression changes of the 9 candidate genes in response to ESAT6 or CFP10 stimuli in vitro for 48 hours. (a) *PARP9* gene was up-regulated and (b) *miR-505* gene was down-regulated in response to either ESAT6 or CFP10 stimuli. (c)

RASGRP4 gene was up-regulated only in response to CFP10 stimuli. (d) *GNG12* was up-regulated in response to either ESAT6 or CFP10 stimuli. (e) *WIPI2* and (f) *FOXO3* genes were both up-regulated only in response to ESAT6 stimuli. (g) *MRPS18B* and (h) *RPTOR* genes were both up-regulated in response to either ESAT6 or CFP10 stimuli. (i) *CCR6* gene was up-regulated only in response to ESAT6 stimuli.

Figure S1

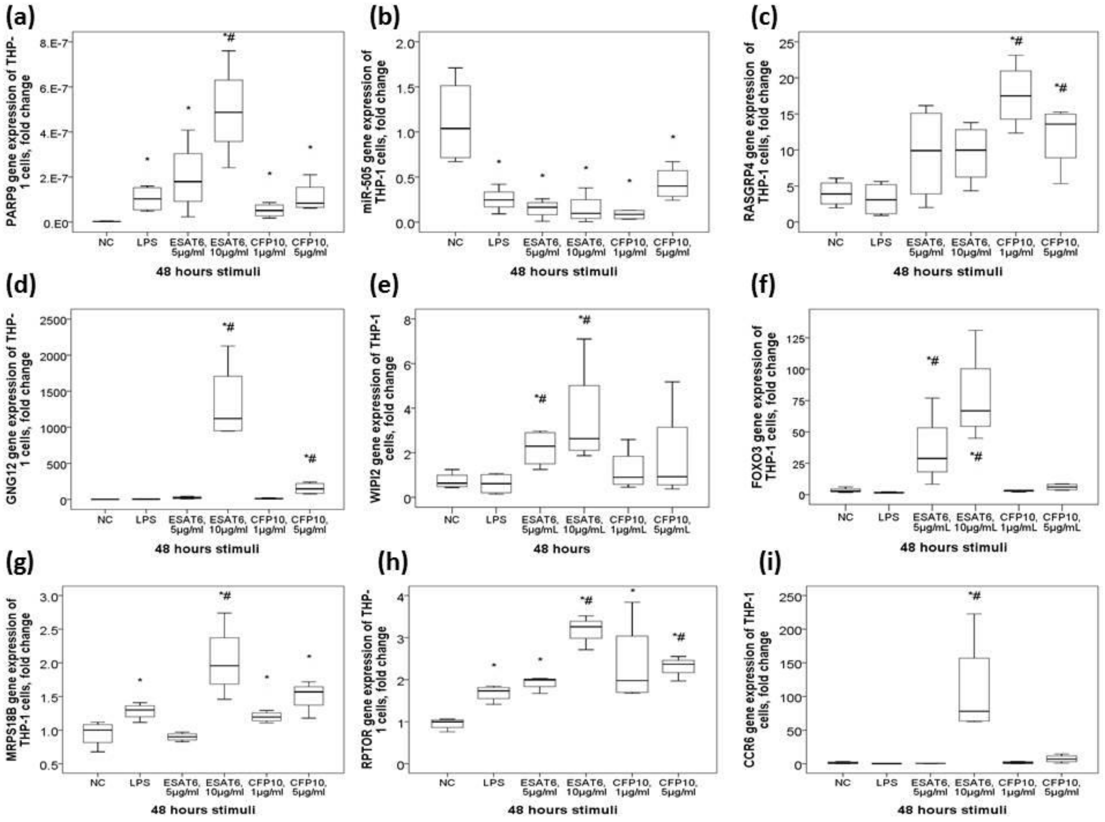
(a)
PARP9 gene expression of THP-1 cells, fold change
48 hours stimuli
(b)
miR-505 gene expression of THP-1 cells, fold change
48 hours stimuli
(c)
RASGRP4 gene expression of THP-1 cells, fold change
48 hours stimuli
(d)
GNG12 gene expression of THP-1 cells, fold change
48 hours stimuli
(e)
WIPI2 gene expression of THP-1 cells, fold change
48 hours
(f)
FOXO3 gene expression of THP-1 cells, fold change
48 hours stimuli
(g)
MRPS18B gene expression of THP-1 cells, fold change
48 hours stimuli
(h)
RPTOR gene expression of THP-1 cells, fold change
48 hours stimuli
(i)
CCR6 gene expression of THP-1 cells, fold change
48 hours stimuli
NC
LPS
ESAT6, 5μg/ml
ESAT6, 10μg/ml
CFP10, 1μg/ml
CFP10, 5μg/ml
